# The first identified cathelicidin from tree frogs possesses anti-inflammatory and partial LPS neutralization activities

**DOI:** 10.1007/s00726-017-2449-7

**Published:** 2017-06-07

**Authors:** Lixian Mu, Lei Zhou, Juanjuan Yang, Li Zhuang, Jing Tang, Tong Liu, Jing Wu, Hailong Yang

**Affiliations:** 1 School of Basic Medical Sciences, Kunming Medical University, 1168 West Chunrong Road, Yuhua Avenue, Chenggong District, Kunming, 650500 Yunnan China; 2 Department of Ear-Nose-Throat, The 184th Hospital of People’s Liberation Army, Yingtan, Jiangxi China; 3 Institute of Pharmaceutical Biotechnology and Engineering, College of Biological Science and Biotechnology, Fuzhou University, Fuzhou, Fujian China; 4 The Second Department of Internal Medicine, The Third Affiliated Hospital of Kunming Medical University, Kunming, Yunnan China

**Keywords:** Cathelicidin, Anti-inflammation, LPS neutralization, Tree frog, *Polypedates puerensis*

## Abstract

As of February 2017, approximately 7639 amphibian species have been described in the AmphibiaWeb database. However, only 20 cathelicidin-like antimicrobial peptides have been identified to date from 10 amphibian species. Half of these peptides were identified from genome sequences and have not yet been functionally characterized. In this study, a novel cathelicidin-like peptide designated cathelicidin-PP was purified from the skin of tree frog *Polypedates puerensis*. Cathelicidin-PP is a 32 residue peptide of sequence ASENGKCNLLCLVKKKLRAVGNVIKTVVGKIA. Circular dichroism spectroscopy indicated that cathelicidin-PP mainly adopts a β-sheet structure in membrane-mimetic solutions. Cathelicidin-PP exhibits potent antimicrobial activity against bacteria and fungi, especially Gram-negative bacteria. Meanwhile, it shows low cytotoxicity toward mammalian cells. Scanning electron microscopy analysis indicated that cathelicidin-PP kills bacteria through the disruption of the bacterial cell membrane integrity. Furthermore, cathelicidin-PP exerts significant anti-inflammatory functions by inhibiting the lipopolysaccharide (LPS)-mediated generation of nitric oxide and pro-inflammatory cytokines, tumor necrosis factor-α, interleukin-1β, and interleukin-6. The MAPKs (ERK, JNK, and p38) and NF-κB signaling pathways are involved in the anti-inflammatory effect. Cathelicidin-PP caused partial neutralization of LPS in a dose-dependent manner. Quantitative PCR indicated that infection of tree frogs with bacteria causes increased expression of cathelicidin-PP in immune-related tissues. Taken together, cathelicidin-PP is the first identified cathelicidin-like peptide from tree frogs. Our findings demonstrate that in addition to direct bactericidal capacity, cathelicidin-PP also possesses immunomodulatory properties, including partial neutralization of LPS, and inhibiting the production of inflammatory cytokines.

**Electronic supplementary material:**

The online version of this article (doi:10.1007/s00726-017-2449-7) contains supplementary material, which is available to authorized users.

## Introduction

Antimicrobial peptides (AMPs) are a conserved component of the innate immune system which plays an important role in defense against invading microorganisms, as well as in the initiation of inflammation. In animals, AMPs are predominantly expressed in the skin and wet mucosal surfaces, such as the oral cavity, lungs, intestine, eyes, and the genito-urinary tract (Bals et al. 1998; Malm et al. 2000; Murakami et al. 2004, 2005), where they create a powerful chemical shield to maintain the balance between bacterial pathogenicity and host resistance. Cathelicidins are one of the best characterized classes of AMPs in mammals (Burton and Steel 2009). The structure of the precursor includes an N-terminal signal peptide (29–30 amino acid residues), a highly conserved cathelin domain (98–114 residues), and a highly variable C-terminal domain (12–100 residues) (Agier et al. 2015). Cathelicidins have multifaceted roles in immune modulation and inflammation. For example, in addition to exhibiting protective capabilities against a diverse range of pathogens, the human cathelicidin (LL-37) and porcine cathelicidin (PR-39) can induce chemotaxis of certain cell types including neutrophils, monocytes, macrophages, eosinophils, and mast cells (Huang et al. 1997; Tjabringa et al. 2006) and promote wound healing and angiogenesis (Koczulla et al. 2003; Steinstraesser et al. 2008). However, there is relatively limited understanding of the structure and activity relationship for cathelicidins in amphibian (Anura, Caudata, and Gymnophiona).

Amphibians are an ancient group of animals. The AmphibiaWeb database (http://www.amphibiaweb.org/) currently contains 7639 amphibian species (Feb 27, 2017). Amphibians are by far the most important natural source for discovering antimicrobial peptides. Currently, more than 1900 AMPs from 178 species belonging to 28 genera in amphibian skin have already been reported (Xu and Lai 2015), accounting for over 63% of the total number of AMPs (~3000) identified from microorganisms to plants and animals (Agier et al. 2015; Wang et al. 2015). However, only 20 AMPs belonging to the cathelicidin family (17 sequences retrieved from the NCBI databases and 3 sequences extracted from the literature; Sun et al. 2015; Yu et al. 2013) have been identified in amphibian. Among these, ten sequences were identified by genome analysis from the Tibetan Plateau frog *Nanorana parkeri* (Anura: Ranidae), the western clawed frog *Xenopus tropicalis* and African clawed frog *Xenopus laevis* (Anura: Pipidae); six sequences [cathelicidin-RC1 and -RC2 (Ling et al. 2014), Lf-CATH1 and -2 (Yu et al. 2013), cathelicidin-AL (Hao et al. 2012), and cathelicidin-PY (Wei et al. 2013)] were identified from four species (*Rana catesbeiana*, *Limnonectes fragilis*, *Amolops loloensis,* and *Paa yunnanensis*) of ranid frogs; three sequences (cathelicidin-DM, BG-CATH, and cathelicidin-Bg) were identified from two species (*Duttaphrynus melanostictus* and *Bufo bufo gargarizans*) of bufonid toads (Gao et al. 2016; Sun et al. 2015); one sequence (tylotoin) was identified from the salamander *Tylototriton verrucosus* (Caudata: Salamandridae) (Mu et al. 2014).

Cathelicidins have been described in a variety of vertebrate species, ranging from fish, reptiles and birds, to mammals (Zanetti 2005). Based on the inherent number of cathelicidin genes, cathelicidins can be used to segregate vertebrate species into two groups: “monocathelicidin species”, which contain a sole cathelicidin gene, and “polycathelicidin species”, which possess different gene-clusters encoding different cathelicidins (Sang et al. 2007). Ten genome-based cathelicidins in three species of frogs and previous study on amphibian cathelicidins (Ling et al. 2014; Yu et al. 2013) indicate that the anuran frogs are “polycathelicidin species”. According to the data from AmphibiaWeb, 964 amphibian species belonging to the family Hylidae have been described so that these frogs may produce more than 1928 cathelicidins. However, no cathelicidin-like peptide has been identified and characterized from hylid frogs, a group of special amphibian species which mainly adopt an arboreal way of life.

We report herein the purification and characterization of a novel cathelicidin-like antimicrobial peptide (cathelicidin-PP) from the skin of tree frog *Polypedates puerensis*. We examined the anti-inflammatory effect of cathelicidin-PP in mouse peritoneal macrophages, and the LPS-induced inflammatory signaling pathways were also studied. In addition, we showed the induction of cathelicidin-PP mRNA in immune- related tissues after tree frogs were challenged with *Escherichia coli*.

## Materials and methods

### Peptide purification

**Fig. 1:**
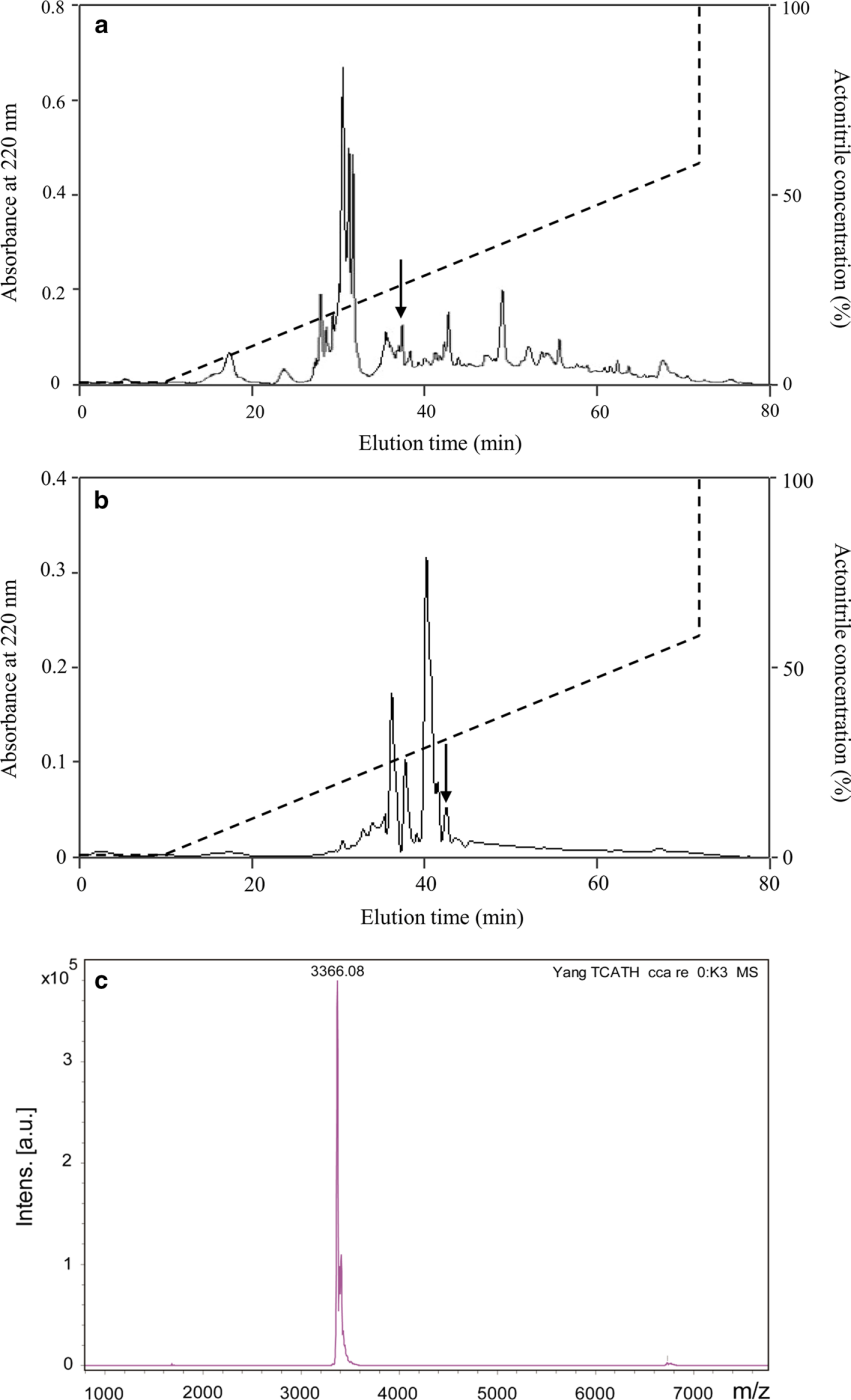
Purification of cathelicidin-PP from the skin of *P. puerensis* and MALDI–TOF MS. **a** The filtrate of the skin secretion of *P. puerensis* by 10 kDa cutoff was divided by a Wondasil C_18_ RP-HPLC column. **b** The eluted peak (*arrow* in **a**) containing antimicrobial activity was further purified by C_18_ RP-HPLC column. The purified cathelicidin-PP is indicated by an *arrow.* **c** MALDI-TOF mass spectrometry analysis of cathelicidin-PP

According to our previous peptide purification procedures of tree frog secretions (Wei et al. 2015), the eluted peak (arrow in Fig. 1a) possessing antimicrobial activity was further purified by RP-HPLC on a Wondasil C_18_ column (25 × 0.46 cm). The elution was performed using a linear gradient of 0–60% acetonitrile containing 0.1% (v/v) trifluoroacetic acid over 70 min (Fig. 1b). The eluted fractions were collected and their antimicrobial activity tested.

### Primary structural analysis

N-terminal sequence of the purified peptide was determined by Edman degradation on an Applied Biosystems pulsed liquid-phase sequencer (model ABI 491). Matrix-assisted laser desorption/ionization time-of-flight mass spectrometry (MALDI-TOF MS) was used to identify the purified peptide. AXIMA CFR mass spectrometer (Kratos Analytical) was analyzed in linear and positive ion mode using an acceleration voltage of 20 kV and an accumulating time of single scanning of 50 s.

### cDNA cloning

The experiment was carried out as described in our previous work (Wei et al. 2015). An In-Fusion SMARTerTM Directional cDNA Library Construction Kit was used for cDNA synthesis. According to the sequence determined by Edman degradation, an antisense degenerate primer (cathelicidin-PP-R_1_) was designed and coupled with a 5′ PCR primer (the adaptor sequence of 3′ In-Fusion SMARTer CDS Primer provided in the kit) to screen the 5′ fragment of cDNA encoding cathelicidin-PP. Then, a sense primer (cathelicidin-PP-F_1_) was designed according to the 5′ coding region and coupled with 3′ PCR primer from the library kit to screen the full-length cDNAs. The PCR conditions were as follows: 95 °C for 5 min and 30 cycles of 95 °C (30 s), 56 °C (45 s), 72 °C (90 s), followed by an extension step at 72 °C for 10 min. Primers used in this research are listed in the supplementary material Table S1.

### Sequence analysis

The theoretical molecular weight of cathelicidin-PP was analyzed through Bioinformatics Resource Portal (http://www.expasy.org/tools/) (Bjellqvist et al. 1993). The assembled sequences were aligned using ClustalW (http://embnet.vital-it.ch/software/ClustalW.html) (Chenna et al. 2003). The phylogenetic tree was constructed by the neighbor-joining method in Mega 6 package (Tamura et al. 2013).

### Antimicrobial assay

Antimicrobial activity of cathelicidin-PP was assayed according to our previous methods (Wei et al. 2015). Microorganisms were obtained from the First Affiliated Hospital of Kunming Medical University. The growth of the microbe was determined by monitoring the absorbance at 600 nm. The minimal concentrations at which no microbial growth occurred were recorded as MIC values.

### Scanning electron microscopy (SEM)

Scanning electron microscopy was performed to evaluate the effect of cathelicidin-PP on the membrane morphology of the bacteria *E. coli* ATCC 25922. The cells were incubated with cathelicidin-PP (1 × MIC) and diluted in phosphate-buffered saline (PBS) at 37 °C for 45 min. After a centrifugation at 1000×*g* for 10 min, the pellets were fixed with 2.5% glutaraldehyde solution for 2 h at 4 °C and then postfixed in 1% osmium tetroxide for 2 h. All specimens were dehydrated through a graded series of alcohols. After the specimens were mounted onto aluminum stubs and sputtered with gold, the SEM images were taken on a Hitachi S-4800 electron microscope.

### Circular dichroism (CD) spectroscopy

Circular dichroism spectroscopy was performed to estimate the secondary structural elements of cathelicidin-PP in membrane-mimetic environments. CD spectra were collected on a Jasco-810 spectropolarimeter (Jasco, Tokyo, Japan) with a 1-mm path-length cell at 25 °C and 0.2-nm intervals from 190 to 260 nm. Cathelicidin-PP was dissolved in H_2_O, sodium dodecyl sulfate (SDS)/H_2_O solutions or LPS/H_2_O solutions to an ultimate concentration of 0.2 mg/ml. The data from three scans were averaged using the Jasco-810 software for each spectrum. CD data were expressed as the mean residue ellipticity (*θ*) in deg cm^2^/dmol.

### Hemolysis and cytotoxicity

Hemolytic assay was performed according to the method described in our previous work (Wei et al. 2015). Rabbit erythrocyte suspensions were incubated with cathelicidin-PP and then the absorbance of supernatant was measured at 540 nm. 1% (v/v) Triton X-100 and PBS were used as positive and negative controls, respectively.

Cytotoxicity against mouse peritoneal macrophages was determined by the MTT assay. Peritoneal macrophages from C57BL/6 mice were prepared as described in our previous work (Wu et al. 2015). Cathelicidin-PP dissolved in serum-free RPMI 1640 medium was added to mouse peritoneal macrophages in 96-well plates (2 × 10^4^ cells/well), and the serum-free RPMI 1640 medium without cathelicidin-PP was used as control. After incubation for 24 h, 20 μl of MTT solution (5 mg/ml) was added to each well, and the cells were further incubated for 4 h. Finally, cells were dissolved in 200 μl of Me_2_SO, and the absorbance at 570 nm was measured.

### Nitric oxide (NO) detection

Mouse peritoneal macrophages were cultured in 24-well plates (2.5 × 10^5^ cells/well). The cells were incubated for 24 h either with LPS (100 ng/ml, from *E. coli* 0111:B4, Sigma-Aldrich, USA) and cathelicidin-PP (0, 5, 10, and 20 μg/ml) or incubated with cathelicidin-PP (10 μg/ml) alone. The culture medium was harvested to detect the nitrite level using Griess reagent (Beyotime, China) according to the manufacturer’s instructions.

### Quantitative PCR (qPCR)

Mouse peritoneal macrophages were cultured in 6-well plates (2 × 10^6^ cells/well) with RPMI 1640 (2% FBS). The cells were incubated either with LPS (100 ng/ml) and cathelicidin-PP (0, 5, 10, and 20 μg/ml) or incubated with cathelicidin-PP (10 μg/ml) alone. After treatment for 6 h, the cells were collected and total RNA was isolated. qPCR was performed on a Realplex Mastercycler real-time PCR system (Eppendorf, Germany). The cycle counts of the target genes were normalized to the *β*-*actin* gene, and accordingly the fold changes of the target genes were calculated. The primers used for qPCR are listed in the supplementary material Table S1.

### Pro-inflammatory cytokine determination

Mouse peritoneal macrophages were cultured in 24-well plates (2.5 × 10^5^ cells/well).

The cells were incubated for 6 h either with LPS (100 ng/ml) and cathelicidin-PP (0, 5, 10, and 20 μg/ml) or incubated with cathelicidin-PP (10 μg/ml) alone. The cell culture supernatants were collected and assessed for tumor necrosis factor-α (TNF-α), interleukin-1β (IL-1β), and interleukin-6 (IL-6) by using ELISA kits (Dakewei, China).

### Western blot analysis

Mouse peritoneal macrophages were cultured in 6-well plates (2 × 10^6^ cells/well). The cells were incubated for 30 min either with LPS (100 ng/ml) and cathelicidin-PP (0, 5, 10, and 20 μg/ml) or incubated with cathelicidin-PP (10 μg/ml) alone. The cells were washed twice with ice-cold PBS and lysed with RIPA lysis buffer (Beyotime, China). Then the cytoplasmic or nuclear proteins were extracted for Western blot analysis according to our previously described method (Wu et al. 2010). Primary antibodies of phospho- ERK/ERK, phospho-JNK/JNK, phospho-p38/p38, NF-κBp65 (1:2000, Cell Signaling Technology, USA), and β-actin (1:5000, Santa Cruz Biotechnology, USA) were used in Western blot analysis.

### LPS neutralization assay

The ability of cathelicidin-PP to neutralize LPS was assayed by the *Limulus* amebocyte lysate (LAL) test according to the manufacturer’s instruction (GenScript, Nanjing, China). Briefly, cathelicidin-PP (0, 5, 10, 20, 40 μg/ml) dissolved in PBS were incubated with LPS. After incubation for 30 min, 100 μl of LAL solution was added to LPS-cathelicidin-PP solutions (100 μl) in a pyrogenfree tube and followed by addition of pre-warmed substrate. After incubation for 6 min, the absorbance was measured at 545 nm. The percentage of LPS neutralization was calculated as (*A*_blank_ − *A*_sample_)/*A*_blank_ × 100, where *A*_blank_ represents the absorbance of blank control (50 μl of LAL water + 50 μl of LPS solution).

### Bacterial challenge

This experiment was carried out as previously described (Wei et al. 2015). In brief, both sexes of tree frogs *P. puerensis* (22–28 g, *n* = 5 in each group) captured in Yunnan Province of China (24.786°N 101.362°E) were injected intraperitoneally (50 μl/tree frog) with *E. coli* ATCC 25922 (1 × 10^6^ CFU/ml PBS) or PBS. The immune-related tissues (skin, spleen, gut, and lung) were collected at 6, 12, 24, and 48 h after bacterial challenge. qPCR was performed to analyze the expression of cathelicidin-PP mRNA as described above, with the housekeeping gene *β*-*actin* as an endogenous control. The study was approved by the Animal Care and Use Ethics Committee of Kunming Medical University.

### Statistical analysis

Statistical analysis was performed using GraphPad Prism 5.0 (GraphPad Software Inc., San Diego, CA, USA) and Stata 10.0 software (Stata Corporation, College Station, TX, USA). Data were presented as mean ± SEM and compared using two-tailed equal variance Student’s *t* test. *p < 0.05 and **p < 0.01 were considered statistically significant.

## Results

### Characterization of cathelicidin-PP

**Fig. 2:**
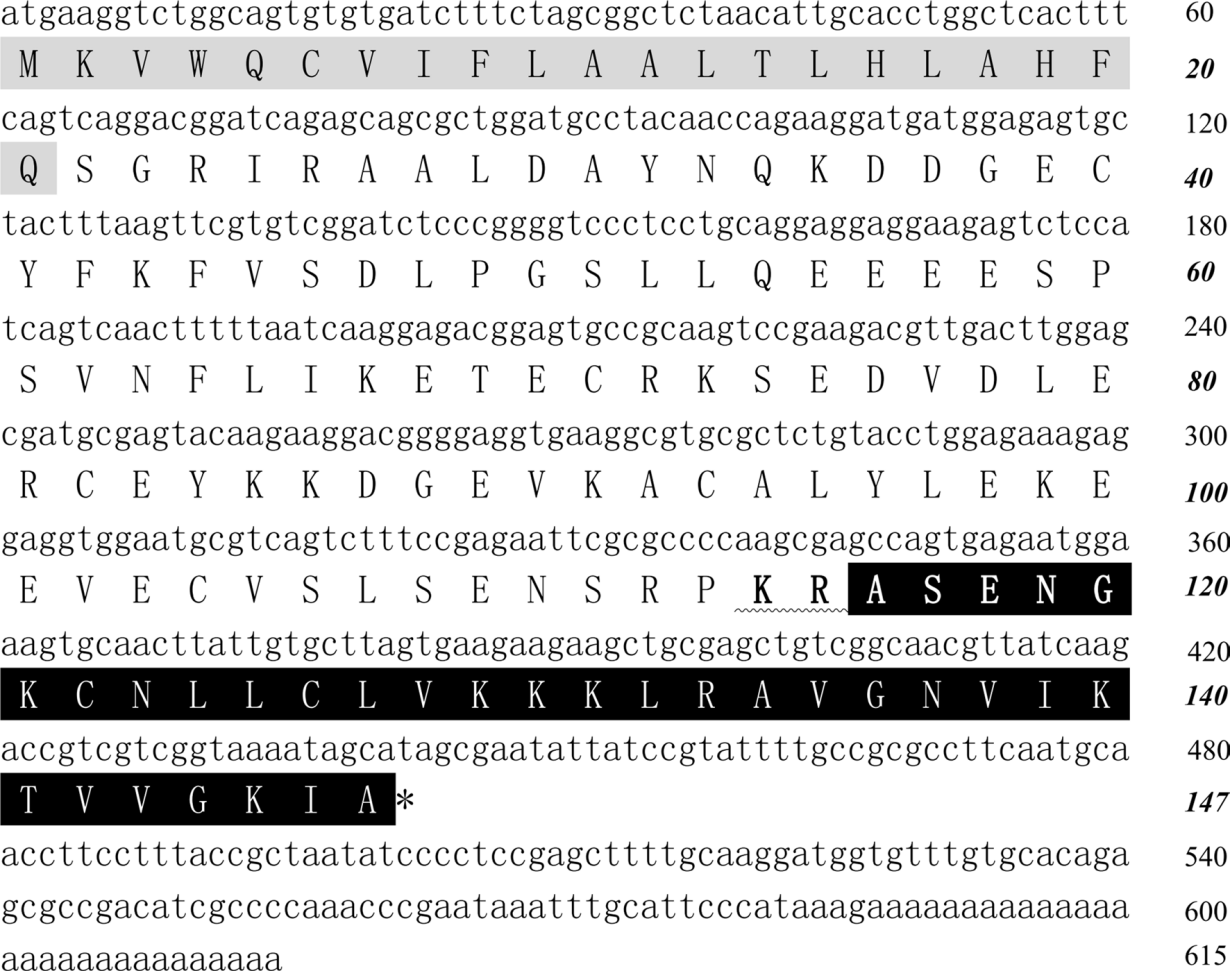
The cDNA sequence of cathelicidin-PP precursor. Deduced amino acid sequence is shown below the cDNA sequence. The cathelicidin-PP precursor contains the signal peptide (*gray*), followed by a cathelin domain ending with a pair of basic residues (in *bold*), and the mature peptide (*black*). The stop codon is indicated by an *asterisk*. Amino acid numbers or nucleotide numbers are shown after the sequences

**Fig. 3:**
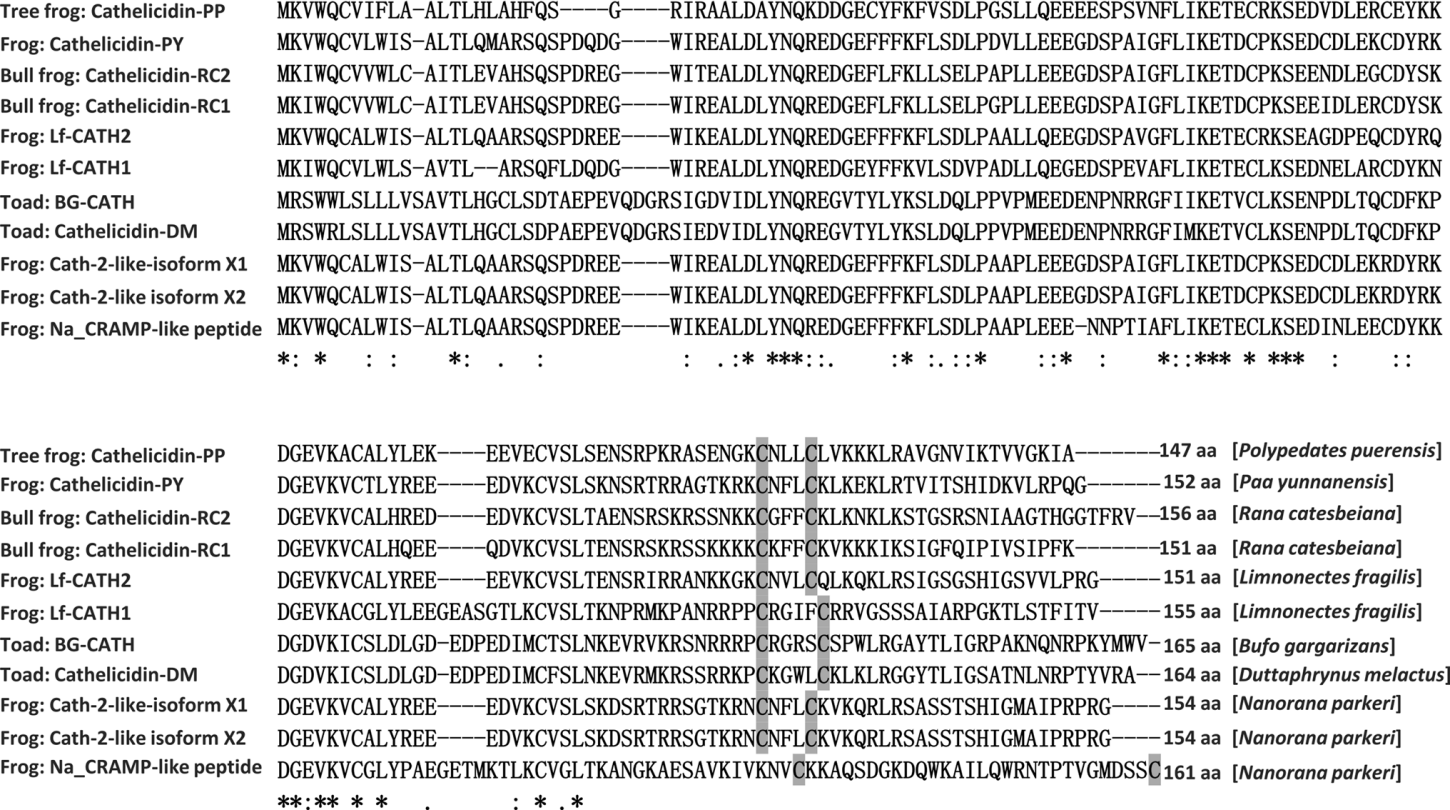
Multi-sequence alignment of cathelicidin-PP precursor with other amphibian cathelicidins. The symbols under the alignment indicate the following: *asterisk* identical sites; *colon* conserved sites; *dot* less conserved sites. *Dashes* are inserted to optimize the alignment. The two conserved cysteine residues involved in disulfide bridges are *gray shaded*. GenBank accession numbers for the analyzed sequences are shown in Fig. 4

The fraction with antimicrobial activity was further purified by C_18_ RP-HPLC (Fig. 1a) and cathelicidin-pp was purified from this step (Fig. 1b). After Edman degradation, the initial 20 N-terminal amino acid residues of cathelicidin-PP were identified with the following sequence: ASENGKCNLLCLVKKKLRAV. MALDI-TOF MS analysis (Fig. 1c) indicated that cathelicidin-PP had a measured molecular mass of 3366.08 Da, matching well with the calculated molecular mass 3366.15 Da. The cDNA clone encoding the precursor of cathelicidin-PP was screened from the skin cDNA library of *P. puerensis* (GenBank accession number: KY610282). The cathelicidin-PP precursor is composed of 147 amino acid residues, including a predicted 21 amino acid signal peptide and a conserved 94 amino acid cathelin domain, followed by a 32 amino acid mature peptide (Fig. 2). The sequence similarity search with the Basic Local Alignment Search Tool (BLAST) indicated that the precursor is a member of the cathelicidin family AMPs, sharing the highest identity of 59% (87/147) with the cathelicidin-PY from the frog *P. yunnanensis*. Multi-sequence alignment of cathelicidin-PP precursor with other amphibian cathelicidin (Fig. 3) indicated that cathelicidin-PP precursor exhibits a high degree of similarity with the other cathelicidins in the cathelin regions, but they are highly variable in the C-terminal mature region. Additionally, a characteristic feature of all these mature peptides is the presence of one intramolecular disulfide bond.

### Phylogenetic analysis of cathelicidin-PP

**Fig. 4:**
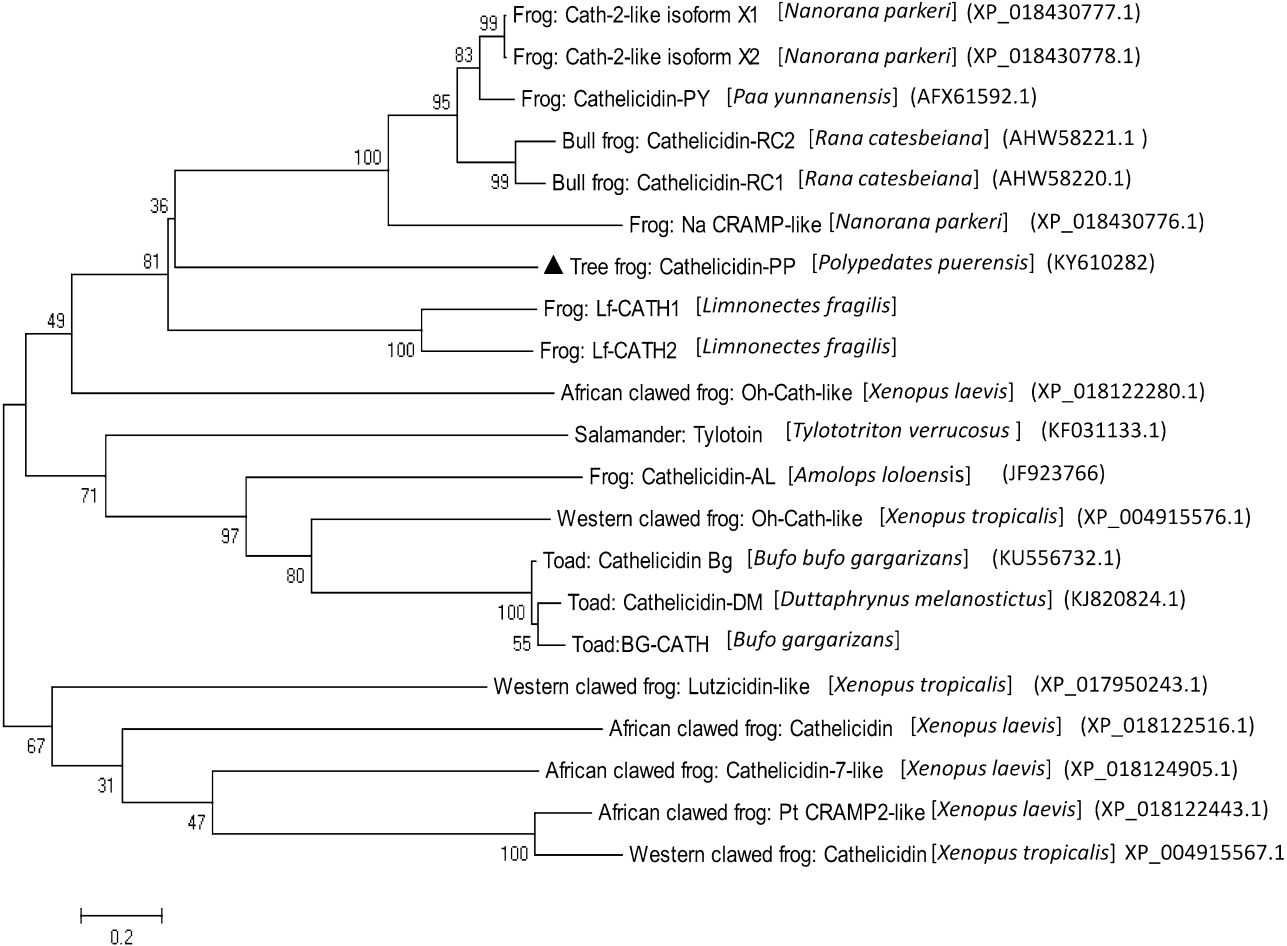
Phylogenetic analysis of known amphibian cathelicidins. The tree was constructed using the neighbor-joining method based on 21 amphibian cathelicidin precursors, including cathelicidin-PP. The *numbers* on the *branches* represent the percent bootstrap support. Cathelicidin-PP is indicated by a *triangle*

The phylogenetic tree was generated from 21 amphibian cathelicidin precursors, which were identified from 11 amphibian species (Fig. 4). The cathelicidin sequences clearly form two distinct clusters: one cluster comprising 5 sequences identified from the clawed frogs (*X. tropicalis* and *X. laevis*) belonging to the Pipidae family, and the second cluster comprising 16 sequences identified from 5 different amphibian families (Pipidae, Ranidae, Bufonidae, Hylidae and Salamandridae). The latter cluster is again divided into two distinct subclusters, in which cathelicidin-PP is grouped together with the eight cathelicidins from four species of ranid frogs (*P. yunnanensis, N. parkeri*, *R. catesbeiana,* and *L. fragilis*).

### Antimicrobial properties of cathelicidin-PP

The antimicrobial potency of cathelicidin-PP against pathogens was determined. As listed in Table 1, cathelicidin-PP exhibited broad-spectrum antimicrobial activities against Gram-positive, Gram-negative bacteria and fungi. In addition, cathelicidin-PP showed much higher antimicrobial activities against Gram-negative bacteria (MICs ranging from 0.69 to 2.78 μM) compared to Gram-positive bacteria (MICs = 5.57 μM). Among the nine strains of the microorganisms tested, the standard strain of *E. coli* displayed the most sensitivity toward cathelicidin-PP, with an MIC value of 0.69 μM.

**Table 1:** Antimicrobial activity of cathelicidin-PP

Microorganisms	MIC (μM)^a^
Gram-negative bacteria	
*Escherichia coli* ATCC 25922	0.69
*Salmonella paratyphi* A ATCC 9150	1.39
*Pseudomonas aeruginosa* ATCC 27853	1.39
*Acinetobacter junii* ATCC 17908	2.78
Gram-positive bacteria	
*Staphylococcus epidermidis* ATCC 12228	5.57
*Staphylococcus haemolyticus* ATCC 29970	5.57
*Enterococcus faecalis* ATCC 29212	5.57
Fungi	
*Candida glabrata* ATCC 66032	1.39
*Candida albicans* ATCC 14053	5.57

*MIC* minimal inhibitory concentration

^a^MICs represent mean values of three independent experiments performed in duplicates

### Cathelicidin-PP alters the morphology of *E. coli*

**Fig. 5:**
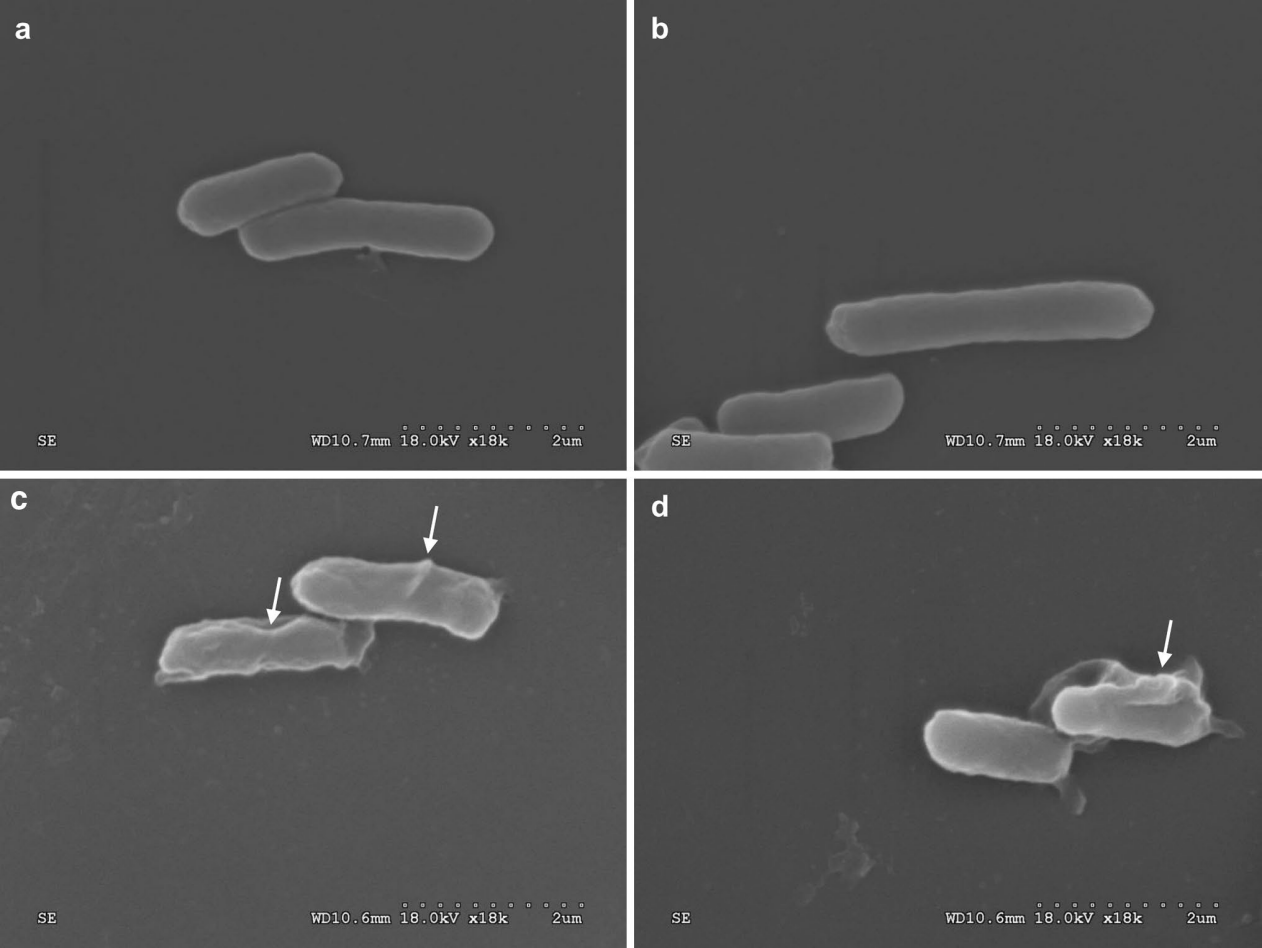
Scanning electron microscopy of bacteria treated with or without cathelicidin-PP. **a**, **b** Control, *E. coli* cells treated with PBS. **c**, **d** *E. coli* cells treated with cathelicidin-PP (1 × MIC, 0.69 μM) dissolved in PBS. *White arrow* indicates damage to the plasma membranes of bacteria or the intracellular inclusions efflux

Scanning electron microscopy was performed to study the effects of cathelicidin-PP on Gram-negative bacteria *E. coli* ATCC25922, which was most sensitive to cathelicidin-PP (Table 1). The control *E. coli* cells exhibited a normal shape and smooth surface (Fig. 5a, b). However, the cells treated with cathelicidin-PP (1 × MIC) showed distinct morphological alterations (Fig. 5c, d). Cathelicidin-PP seemed to disturb the membrane integrity of cells, and cell shrinkage was evident.

### Cathelicidin-PP shows low cytotoxicity and hemolytic activity

Cathelicidin-PP exhibited very low cytotoxicity toward mouse peritoneal macrophages. Cathelicidin-PP induced cell death percentages as low as 7.06% at concentrations up to 200 μg/ml (59.42 μM), which is almost 86-fold higher than the MIC value of cathelicidin-PP against *E. coli* ATCC 25922. Additionally, cathelicidin-PP also showed low hemolytic activity against erythrocytes, and it yielded a hemolysis of 5.65% at the same concentration of 200 μg/ml.

### Secondary structure of cathelicidin-PP

**Fig. 6:**
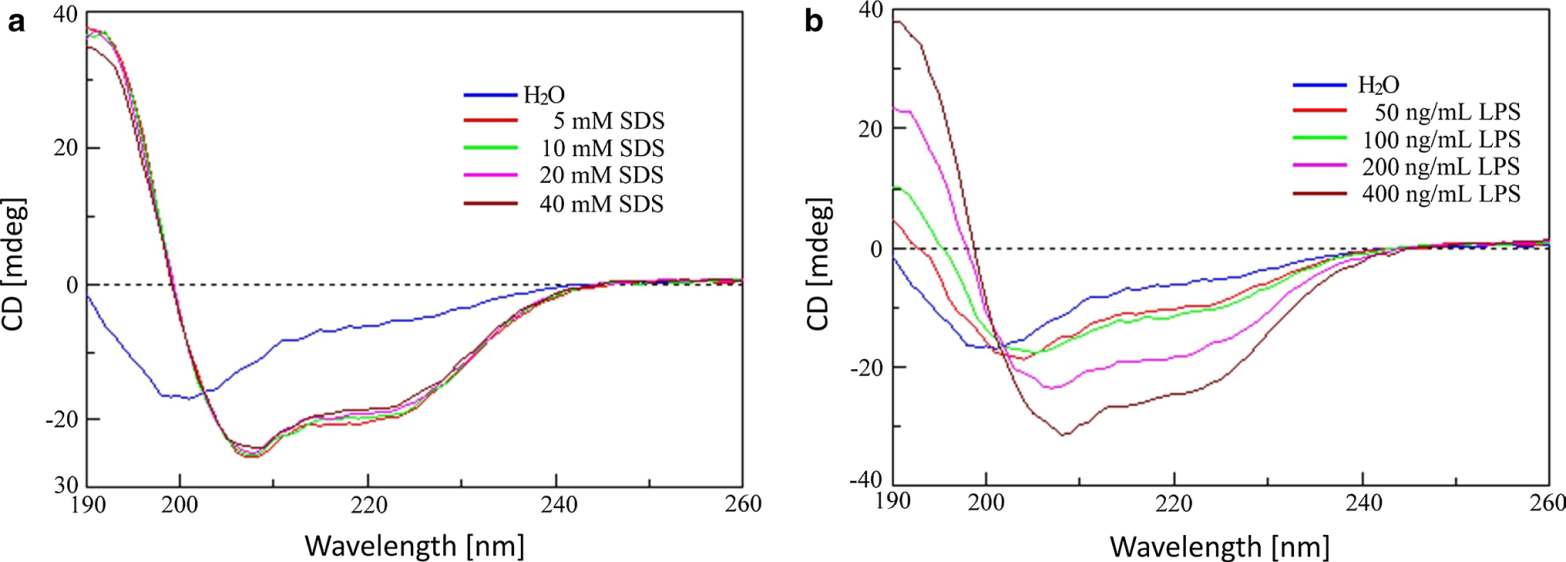
The CD spectra of cathelicidin-PP in different solutions. **a** SDS/H_2_O solution (5, 10, 20, 40 mM). **b** LPS/H_2_O solution (50, 100, 200, 400 ng/ml). Cathelicidin-PP was dissolved in different solutions to an ultimate concentration of 0.2 mg/ml

Each category of secondary structure shows a unique spectrum. The spectral characteristics of α-helices is a positive peak at ~195 nm and two almost equivalent negative peaks at 208 and 222 nm. A small negative peak at ~212 nm and the positive strong peak at 192 nm are the characteristic peaks of β-sheet structure. For random coil structure, there is a negative strong peak at ~198 nm. α-Helical structures from CD spectra are much more well-defined than the β-sheet structures, because the characteristic peaks from α-helices are much larger than the β-sheet contributions (Wallace 2000). The CD spectra of cathelicidin-PP dissolved in H_2_O showed a strong negative peak at 199 nm and a small negative peak at 211 nm (Fig. 6), indicating that it mainly adopted β-sheet and random-coil conformations. In the membrane-mimetic environments of SDS/H_2_O solutions (Fig. 6a), the CD spectra showed that in addition to a small negative peak at 211 nm, there were two inequivalent negative peaks at 208 and 222 nm, indicating that cathelicidin-PP mainly adopted a β-sheet structure with a small α-helix. In addition, with increasing concentration of LPS (Fig. 6b), there were characteristic peaks from helices, which indicated the degree of α-helix content increased as the LPS concentration increased.

As listed in Table 2, the molecular conformation of cathelicidin-PP showed little change in different concentrations of SDS solutions (5, 10, 20, and 40 mM), containing 38.7–40% β-sheet, 25.2–26.5% α-helix, and 34.7–35.6% random coil. However, in various proportions of LPS solutions (50, 100, 200, and 400 ng/ml), cathelicidin-PP displayed a concomitant structural rearrangement which caused a decrease in β-sheet and unordered structures (percent decrease from 53.4 to 47.4, and from 40.9 to 34.2, respectively) but an increase in α-helix (percent increase from 3.8 to 18.4).

**Table 2:** Secondary structural components of cathelicidin-PP in different solutions

Solution	Helix (%)^a^	Beta (%)^a^	Turn (%)^a^	Random (%)^a^
H_2_O	0.0	48.6	5.6	45.8
SDS (mM)				
5	25.4	39.8	0.0	34.8
10	25.2	40.0	0.0	34.8
20	25.7	38.7	0.0	35.6
40	26.5	38.8	0.0	34.7
LPS (ng/ml)				
50	3.8	53.4	1.9	40.9
100	7.0	53.5	0.0	39.5
200	14.0	50.3	0.0	35.7
400	18.4	47.4	0.0	34.2

^a^Jasco-810 software was used to deconvolute CD spectra into fractional contents and these data are the average value of three scans

### Cathelicidin-PP reduces LPS-induced iNOS transcription and NO production

**Fig. 7:**
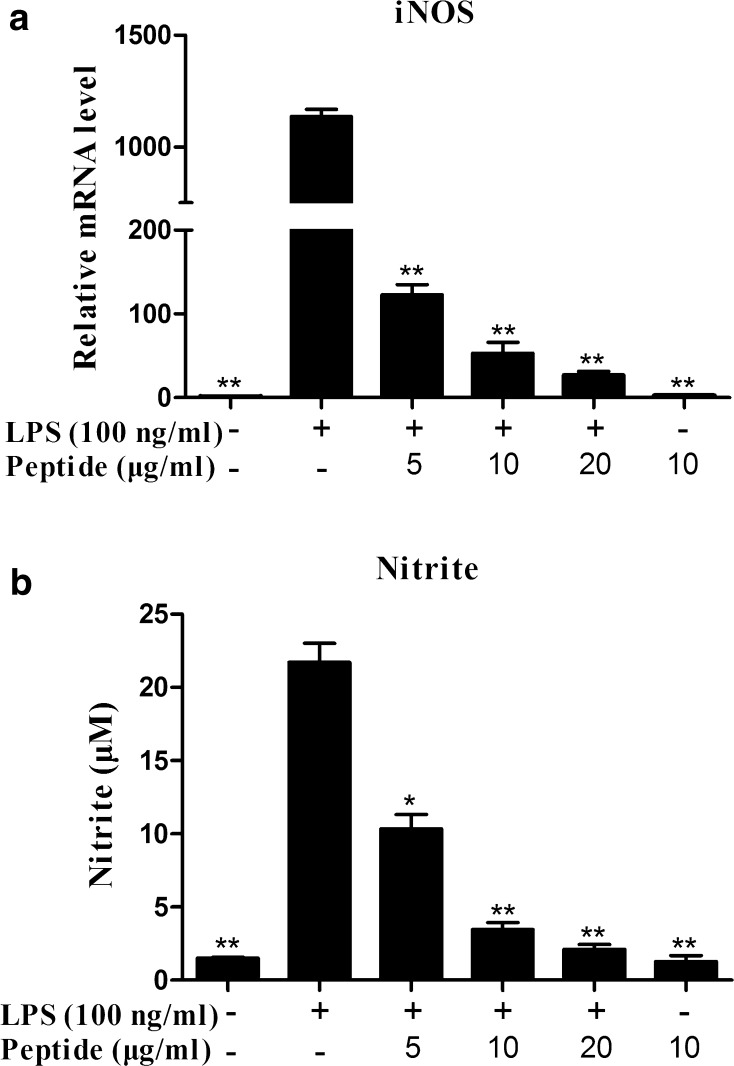
Effects of cathelicidin-PP on iNOS transcription and NO production induced by LPS. **a** iNOS mRNA. **b** Nitrite production. Data are mean ± SEM. Values from three independent experiments. *p < 0.05, **p < 0.01, significantly different compared with the control that was treated with serum-free RPMI 1640 and 100 ng/ml LPS

To evaluate the effect of cathelicidin-PP on the LPS-induced NO production in mouse peritoneal macrophages, the levels of iNOS mRNA were determined by qPCR. Incubation with cathelicidin-PP for 6 h significantly reduced the iNOS mRNA levels induced by 100 ng/ml LPS in a dose-dependent fashion (Fig. 7a). At a concentration of 20 μg/ml, cathelicidin-PP inhibited 97.5% of the iNOS transcription. Furthermore, we determined the NO production by examining the nitrite concentration in the culture supernatants of mouse peritoneal macrophages. The addition of cathelicidin-PP significantly reduced LPS-induced nitrite production (Fig. 7b). At the concentration of 20 μg/ml, cathelicidin-PP inhibited 90.2% of nitrite production.

### Cathelicidin-PP inhibits LPS-induced pro-inflammatory cytokine production

**Fig. 8:**
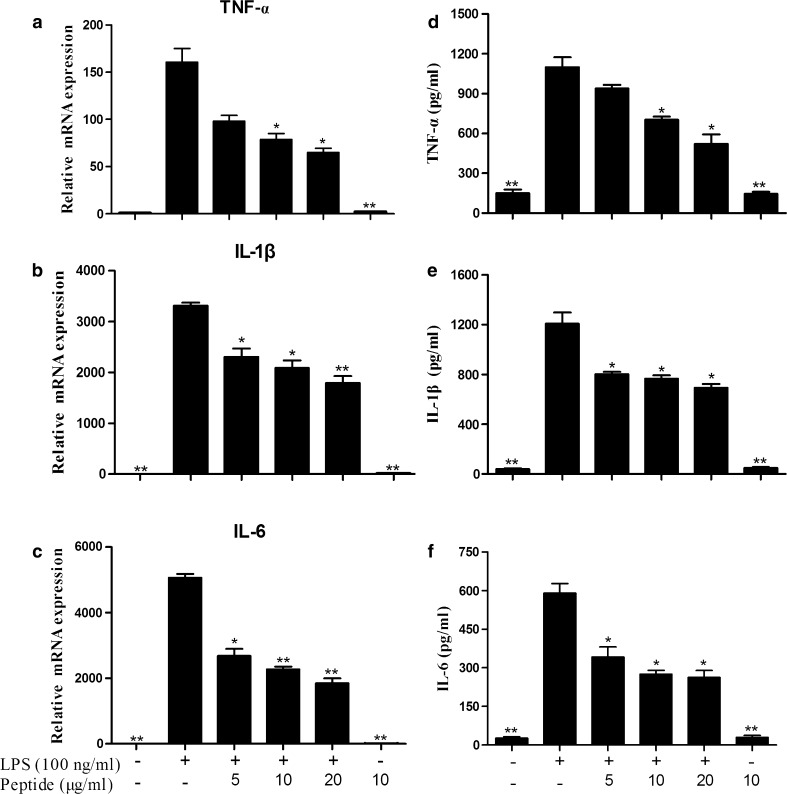
Effects of cathelicidin-PP on pro-inflammatory cytokine transcription and secretion induced by LPS. **a** TNF-α mRNA. **b** TNF-α secretion. **c** IL-1β mRNA. **d** IL-1β secretion. **e** IL-6 mRNA. **f** IL-6 secretion. Data are mean ± SEM. Values from three separate experiments. *p < 0.05, **p < 0.01, significantly different compared with the control that was incubated with serum-free RPMI 1640 and 100 ng/ml LPS

To evaluate the effect of cathelicidin-PP on LPS-induced pro-inflammatory cytokine production in mouse peritoneal macrophages, we first used qPCR to determine proinflammatory cytokine gene expression. Cathelicidin-PP significantly blocked LPS-induced expression of TNF-α, IL-1β, and IL-6 in a dose-dependent manner. 20 μg/ml cathelicidin-PP inhibited the expression of all three of the pro-inflammatory cytokine genes by 59.5, 45.9, and 63.5%, respectively (Fig. 8a–c). Furthermore, we used ELISA to confirm the effect of cathelicidin-PP on pro-inflammatory cytokine production induced by LPS in mouse peritoneal macrophages. Cathelicidin-PP showed activities similar to those obtained in the qPCR experiments. 20 μg/ml cathelicidin-PP inhibited LPS-induced TNF-α, IL-1β, and IL-6 production by 52.5, 42.7, and 55.6%, respectively (Fig. 8d–f).

### Cathelicidin-PP inhibits LPS-induced inflammatory response pathways

**Fig. 9:**
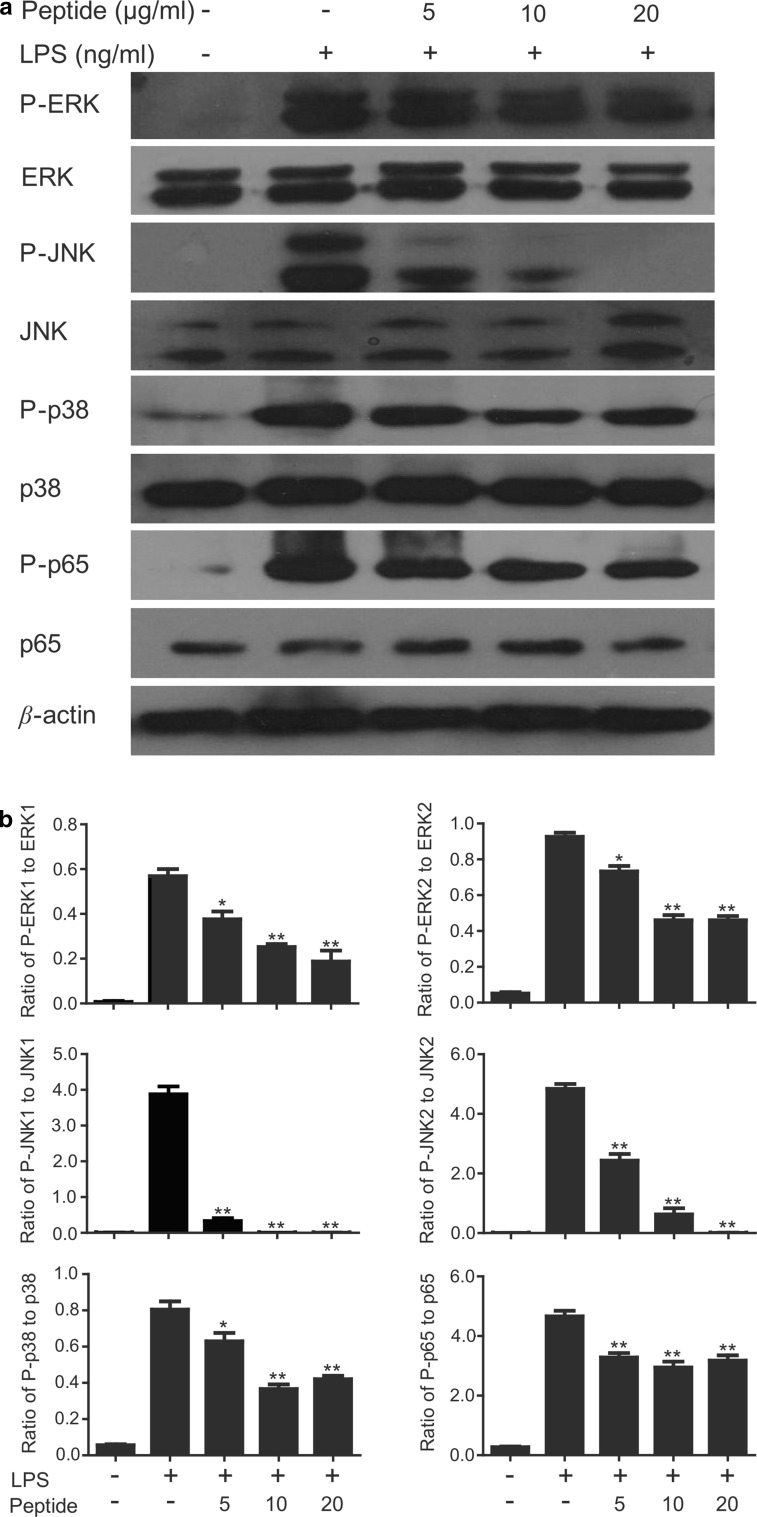
Effects of cathelicidin-PP on LPS-induced inflammatory response pathways. **a** Western blot of phosphorylation of ERK, JNK, p38, and NF-κB p65 in peritoneal macrophages. The cells were incubated with LPS (100 ng/ml) and different concentrations of cathelicidin-PP (0, 5, 10, and 20 μg/ml). After incubation for 30 min, the cells were collected, and the cytoplasmic or nuclear proteins were extracted for Western blot analysis. **b** Ratio of P-ERK1 (44 kDa), P-ERK2 (42 kDa), P-JNK1 (54 kDa), P-JNK2 (46 kDa), P-p38, and P-p65 to β-actin. Band densities were analyzed using Quantity One software (Bio-Rad, Richmond, CA, USA). Data were presented as mean ± SEM. *p < 0.05, **p < 0.01, ratios of peptide-treated groups are significantly different from that induced by 100 ng/ml LPS alone

The above-mentioned data indicate that cathelicidin-PP significantly inhibited the transcription and production of NO and pro-inflammatory cytokines induced by LPS in macrophages. The mitogen-activated protein kinases (MAPKs) and NF-κB signal pathways play important roles in inflammatory responses. Therefore, we studied the effect of cathelicidin-PP on LPS-induced inflammatory signaling pathways. 100 ng/ml LPS significantly induced the phosphorylation of MAPKs (ERK, JNK, and p38) and NF-κB p65. In contrast, the incubation of cathelicidin-PP (5, 10, 20 μg/ml) inhibited the LPS-induced phosphorylation of ERK, JNK, p38, and NF-κB p65, especially JNK (Fig. 9a). At the concentration of 20 μg/ml, cathelicidin-PP inhibited 66.9% P-ERK1, 50.3% P-ERK2, 100% P-JNK1, 100% P-JNK2, 47.8% P-p38, and 31.8% P-p65 expression induced by LPS, respectively (Fig. 9b).

### Cathelicidin-PP partially neutralizes LPS

**Fig. 10:**
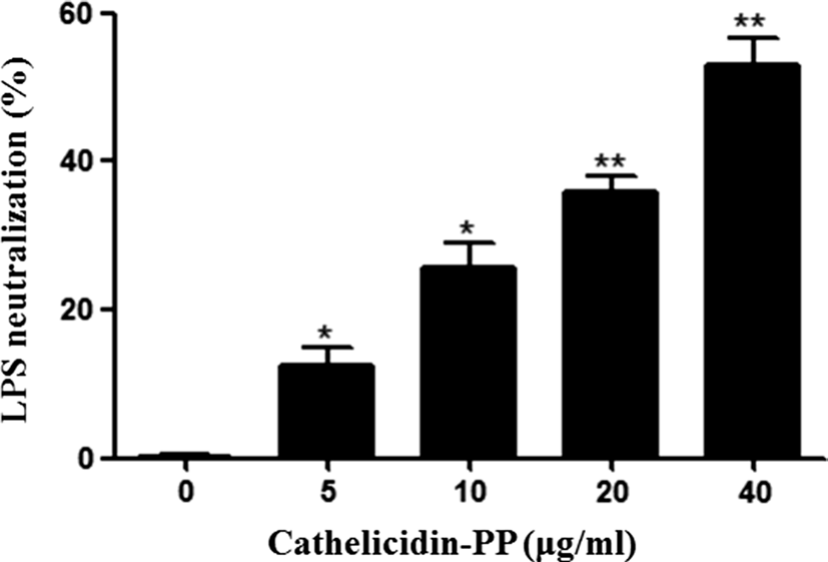
LPS-neutralizing activity of cathelicidin-PP. A chromogenic LAL assay was used to evaluate neutralizing activity. Data are mean ± SEM. Values from three separate experiments. *p < 0.05, **p < 0.01, significantly different compared with the control (PBS)

The chromogenic LAL assay was used to determine whether cathelicidin-PP has a capacity to neutralize endotoxin. Cathelicidin-PP caused partial neutralization of LPS in a dose-dependent manner (Fig. 10). At the concentrations of 5, 10, 20, and 40 μg/ml, cathelicidin-PP inhibited 12.5, 25.7, 35.9, and 53.1% of LPS, respectively.

### Transcript levels of cathelicidin-PP increase after immune challenge

**Fig. 11:**
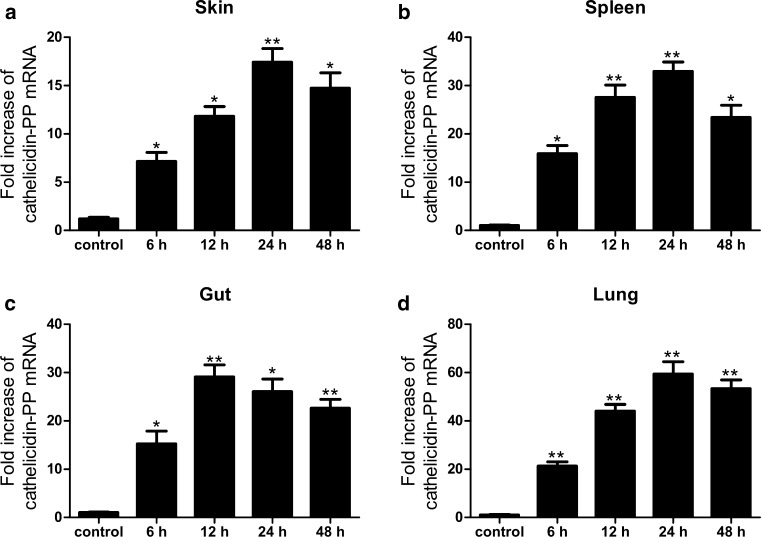
Fold increase of cathelicidin-PP in immune-related tissues at different time courses after immune challenge with *E. coli*. **a** Fold increase of cathelicidin-PP in skin. **b** Fold increase of cathelicidin-PP in spleen. **c** Fold increase of cathelicidin-PP in gut. **d** Fold increase of cathelicidin-PP in lung. Expression levels in different tissues were calculated relative to the level of cathelicidin-PP in corresponding uninfected tissue, which was arbitrarily defined as 1. Values for infection treatment are significantly different from control values. *p < 0.05, **p < 0.01, significantly different compared to the control (*n* = 5)

After infection with *E. coli*, the expression of cathelicidin-PP mRNA was examined at different time courses in the immune-related tissues including skin, gut, lung, and spleen. At 6, 12, 24, and 48 h after *E. coli* injection, the mRNA level of cathelicidin-PP was increased in skin (7.2-, 11.8-, 17.4-, and 14.7-fold, respectively), in spleen (15.9-, 27.9-, 32.9-, and 23.4-fold, respectively), in gut (15.3-, 29.1-, 26.1- and 22.7-fold, respectively) and in lung (21.3-, 44.1-, 59.4- and 53.3-fold, respectively) (Fig. 11). The expression of cathelicidin-PP peaked at 24 h post-injection and relatively decreased with time in these tissues, except for the gut in which cathelicidin-PP mRNA reached maximum levels by 12 h after stimulation.

## Discussion

Amphibians have developed an effective strategy for survival in different habitats (aquatic, semiaquatic, terrestrial, arboreal, torrential, and rocky). The skin secretions of different species contain a large number of biologically active peptides that contribute to the reduction in risk of biotic and abiotic damage (Wu et al. 2011; Yang et al. 2009). Among these peptides, AMPs are a class of well-characterized immune defense molecules. To date, some distinct families of AMPs have been characterized from different species of tree frogs, e.g., dermaseptin (Charpentier et al. 1998), dermatoxin (Amiche et al. 2000), plasticin (Vanhoye et al. 2004), and aurein (Boland and Separovic 2006). These peptides comprise an active defensive barrier in the tree frog skin, but no tree frog cathelicidin antimicrobial peptide has been reported.

The current work identified a novel defensive peptide (cathelicidin-PP) from the skin of the tree frog *P. puerensis*. The structural organization of cathelicidin-PP precursor (Fig. 2) is similar to other vertebrate cathelicidin precursors, comprising a putative signal peptide, a cathelin domain, and the mature peptide. Although elastase, which has a clear preference for valine and alanine, is the enzyme primarily responsible for the processing of the cathelicidin precursor to generate the mature peptide in fish, bird and mammals (Shinnar et al. 2003), cathelicidin-PP shares a characteristic dipeptide cleavage site (–K_114_R_115_–) for trypsin-like proteases between the cathelin domain and mature peptide. The enzymatic processing sites are consistent with the skin secretion AMPs identified from hylid and ranid frogs (Vanhoye et al. 2003). Cathelicidin-PP contains two cysteines that form an intramolecular disulfide bridge. The same structure feature exists in the known amphibian cathelicidins, except for cathelicidin-AL and two predicted cathelicidins (GenBank Accession Numbers: XP_018122516.1 and XP_018124905.1) from African clawed frog *X. laevis*. These three linear cathelicidins are devoid of cysteine, but rich in glycine.

The amino acid sequences of cathelicidins produced by different amphibian species are highly variable and species-specific, which suggests that they may be valuable in taxonomic classification and molecular phylogenetic analysis. The phylogenetic tree of 21 amphibian cathelicidin precursors (Fig. 4) showed that cathelicidin-PP is closely related to the eight cathelicidins from four species of ranid frogs (*P. yunnanensis, N. parkeri*, *R. catesbeiana* and *L. fragilis*). The result could be taken as support for the speculation that the skin secretion AMPs from hylid and ranid frogs arose from a 150-million-year-old ancestral precursor (Vanhoye et al. 2003). In addition, since current data for cathelicidins in amphibians are less well-established, this evolutionary tree does not agree with the traditional classification of amphibians. More efforts should be made to identify and characterize cathelicidin antimicrobial peptides from amphibians.

Cathelicidin-PP is a highly basic peptide with a net charge of +6, which could contribute to the adhesion of the negatively charged bacterial surface by electrostatic attractive force. The antibacterial analysis (Table 1) indicated that cathelicidin-PP possesses potent antibacterial activity against bacteria and fungi, especially Gram-negative bacteria (MICs ranging from 0.69 to 2.78 μM). SEM analysis (Fig. 5) indicated that the lysis of bacterial membranes is at least partially responsible for this activity. Some membrane-lytic peptides are linear, mostly helical, and others possess one or more disulfide bonds forming β-sheet or both β-sheet and α-helix structures (Boland and Separovic 2006). CD spectroscopy indicated that cathelicidin-PP mainly forms a β-sheet conformation with few α-helix in membrane-mimetic solutions (Fig. 6). These results confirm that cathelicidin-PP is a membrane-targeting antimicrobial peptide that acts by increasing the permeability of the microbial membrane.

It is becoming increasingly clear that apart from their direct antimicrobial activity, AMPs have an important role in modulating host immune and inflammatory responses (Bowdish et al. 2005). In this study, cathelicidin-PP significantly inhibited the transcription and production of pro-inflammatory factors induced by LPS in mouse peritoneal macrophages, including NO, TNF-α, IL-1β, and IL-6 (Figs. 7, 8). Furthermore, western blot analysis showed that cathelicidin-PP blocks the activation of MAPKs (ERK, JNK, and p38) and NF-κB signal pathways (Fig. 9). The above results indicate that cathelicidin-PP is not only involved in inhibiting microorganism growth but also that it attenuates inflammatory responses induced by LPS. Furthermore, cathelicidin-PP exerts an anti-inflammatory effect in LPS-stimulated murine macrophages by blocking the activation of MAPKs and NF-κB signaling pathways which have been documented to regulate the transcriptions of cytokine genes (Wei et al. 2013; Wu et al. 2010). Further analysis of interaction between cathelicidin-PP and LPS indicated that cathelicidin-PP can partially neutralize LPS in a dose-dependent fashion (Fig. 10). Taken together, these results suggest that cathelicidin-PP may block the binding of LPS to LPS-binding protein, and hence suppresses the release of cytokines and other inflammatory mediators induced by LPS. Interfering with the ability of LPS to function in immune responses is likely to be an effective mechanism for preventing bacterial infections caused by Gram-negative bacteria and/or their endotoxins (LPS), such as Gram-negative sepsis which is a life-threatening systemic inflammatory response syndrome (Schulte et al. 2013). Although our data indicate that cathelicidin-PP can partially neutralize LPS and inhibit the production of pro-inflammatory cytokines in vitro, further in vivo investigations are required to determine whether cathelicidin-PP has potential in the treatment of endotoxin shock and sepsis associated with bacterial infections. The transcript levels of cathelicidin-PP in immune-related tissues of tree frogs increased after infection with Gram-negative bacteria *E.* *coli*, reaching maximum at 12 h (in gut) or 24 h post-injection (in skin, spleen and lung) and then slowly decreased from that time point (Fig. 11). Together, these data suggest that cathelicidin-PP is involved in the innate response of tree frog *P. puerensis* and it favors resolution of infection and potentially harmful inflammation in vivo.

## Conclusions

In conclusion, cathelicidin-PP is the first cathelicidin-like peptide identified from tree frogs. Cathelicidin-PP is a membrane-targeting peptide with a Lys-rich sequence, exhibiting strong antimicrobial activity against bacteria and fungi. Meanwhile, it shows low cytotoxicity toward mammalian cells. Furthermore, it can partially neutralize LPS and suppress the MAPK- and NF-κB-mediated production of pro-inflammatory cytokines. The combination of these properties makes cathelicidin-PP an attractive candidate for both anti-infective and anti-inflammatory therapeutics.

## Electronic supplementary material

Below is the link to the electronic supplementary material.

Supplementary material 1 (DOC 38 kb)

## References

[CR1] Agier J, Efenberger M, Brzezińska-Błaszczyk E (2015). Cathelicidin impact on inflammatory cells. Cent Eur J Immunol.

[CR2] Amiche M, Seon AA, Wroblewski H, Nicolas P (2000). Isolation of dermatoxin from frog skin, an antibacterial peptide encoded by a novel member of the dermaseptin genes family. Eur J Biochem.

[CR3] Bals R, Wang X, Zasloff M, Wilson JM (1998). The peptide antibiotic LL-37/hCAP -18 is expressed in epithelia of the human lung where it has broad antimicrobial activity at the airway surface. Proc Natl Acad Sci USA.

[CR4] Bjellqvist B, Hughes GJ, Pasquali C, Paquet N, Ravier F, Sanchez JC, Frutiger S, Hochstrasser D (1993). The focusing positions of polypeptides in immobilized pH gradients can be predicted from their amino acid sequences. Electrophoresis.

[CR5] Boland MP, Separovic F (2006). Membrane interactions of antimicrobial peptides from Australian tree frogs. Biochim Biophys Acta.

[CR6] Bowdish DM, Davidson DJ, Scott MG, Hancock RE (2005). Immunomodulatory activities of small host defense peptides. Antimicrob Agents Chemother.

[CR7] Burton MF, Steel PG (2009). The chemistry and biology of LL-37. Nat Prod Rep.

[CR8] Charpentier S, Amiche M, Mester J, Vouille V, Le Caer JP, Nicolas P, Delfour A (1998). Structure, synthesis, and molecular cloning of dermaseptins B, a family of skin peptide antibiotics. J Biol Chem.

[CR9] Chenna R, Sugawara H, Koike T, Lopez R, Gibson TJ, Higgins DG, Thompson JD (2003). Multiple sequence alignment with the Clustal series of programs. Nucleic Acids Res.

[CR10] Gao F, Xu WF, Tang LP, Wang MM, Wang XJ, Qian YC (2016) Characteristics of cathelicidin-Bg, a novel gene expressed in the ear-side gland of *Bufo gargarizans*. Genet Mol Res 15(3). doi:10.4238/gmr.15038481 10.4238/gmr.15038481 27525920

[CR11] Hao X, Yang H, Wei L, Yang S, Zhu W, Ma D, Yu H, Lai R (2012). Amphibian cathelicidin fills the evolutionary gap of cathelicidin in vertebrate. Amino Acids.

[CR12] Huang HJ, Ross CR, Blecha F (1997). Chemoattractant properties of pr-39, a neutrophil antibacterial peptide. J Leukoc Biol.

[CR13] Koczulla R, von Degenfeld G, Kupatt C, Krotz F, Zahler S, Gloe T, Issbrucker K, Unterberger P, Zaiou M, Lebherz C, Karl A, Raake P, Pfosser A, Boekstegers P, Welsch U, Hiemstra PS, Vogelmeier C, Gallo RL, Clauss M, Bals R (2003). An angiogenic role for the human peptide antibiotic ll-37/hcap-18. J Clin Investig.

[CR14] Ling G, Gao J, Zhang S, Xie Z, Wei L, Yu H, Wang Y (2014). Cathelicidins from the bullfrog *Rana catesbeiana* provides novel template for peptide antibiotic design. PLoS One.

[CR15] Malm J, Sørensen O, Persson T, Frohm-Nilsson M, Johansson B, Bjartell A, Lilja H, Ståhle-Bäckdahl M, Borregaard N, Egesten A (2000). The human cationic antimicrobial protein (hCAP-18) is expressed in the epithelium of human epididymis, is present in seminal plasma at high concentrations, and is attached to spermatozoa. Infect Immun.

[CR16] Mu L, Tang J, Liu H, Shen C, Rong M, Zhang Z, Lai R (2014). A potential wound- healing-promoting peptide from salamander skin. FASEB J.

[CR17] Murakami M, Lopez-Garcia B, Braff M, Dorschner RA, Gallo RL (2004). Postsecretory processing generates multiple cathelicidins for enhanced topical antimicrobial defense. J Immunol.

[CR18] Murakami M, Dorschner RA, Stern LJ, Lin KH, Gallo RL (2005). Expression and secretion of cathelicidin antimicrobial peptides in murine mammary glands and human milk. Pediatr Res.

[CR19] Sang Y, Teresa Ortega M, Rune K, Xiau W, Zhang G, Soulages JL, Lushington GH, Fang J, Williams TD, Blecha F, Melgarejo T (2007). Canine cathelicidin (K9CATH): gene cloning, expression, and biochemical activity of a novel pro-myeloid antimicrobial peptide. Dev Comp Immunol.

[CR20] Schulte W, Bernhagen J, Bucala R (2013). Cytokines in sepsis: potent immunoregulators and potential therapeutic targets-an updated view. Mediat Inflamm.

[CR21] Shinnar AE, Butler KL, Park HJ (2003). Cathelicidin family of antimicrobial peptides: proteolytic processing and protease resistance. Bioorg Chem.

[CR22] Steinstraesser L, Koehler T, Jacobsen F, Daigeler A, Goertz O, Langer S, Kesting M, Steinau H, Eriksson E, Hirsch T (2008). Host defense peptides in wound healing. Mol Med.

[CR23] Sun T, Zhan B, Gao Y (2015). A novel cathelicidin from *Bufo bufo gargarizans* Cantor showed specific activity to its habitat bacteria. Gene.

[CR24] Tamura K, Stecher G, Peterson D, Filipski A, Kumar S (2013). MEGA6: molecular evolutionary genetics analysis version 6.0. Mol Biol Evol.

[CR25] Tjabringa GS, Ninaber DK, Drijfhout JW, Rabe KF, Hiemstra PS (2006). Human cathelicidin ll-37 is a chemoattractant for eosinophils and neutrophils that acts via formyl-peptide receptors. Int Arch Allergy Immunol.

[CR26] Vanhoye D, Bruston F, Nicolas P, Amiche M (2003). Antimicrobial peptides from hylid and ranin frogs originated from a 150-million-year-old ancestral precursor with a conserved signal peptide but a hypermutable antimicrobial domain. Eur J Biochem.

[CR27] Vanhoye D, Bruston F, El Amri S, Ladram A, Amiche M, Nicolas P (2004). Membrane association, electrostatic sequestration, and cytotoxicity of gly-leu-rich peptide orthologs with differing functions. Biochemistry.

[CR28] Wallace BA (2000). Synchrotron radiation circular-dichroism spectroscopy as a tool for investigating protein structures. J Synchrotron Radiat.

[CR29] Wang G, Mishra B, Lau K, Lushnikova T, Golla R, Wang X (2015). Antimicrobial peptides in 2014. Pharmaceuticals (Basel).

[CR30] Wei L, Yang J, He X, Mo G, Hong J, Yan X, Lin D, Lai R (2013). Structure and function of a potent lipopolysaccharide-binding antimicrobial and anti- inflammatory peptide. J Med Chem.

[CR31] Wei L, Che H, Han Y, Lv J, Mu L, Lv L, Wu J, Yang H (2015). The first anionic defensin from amphibians. Amino Acids.

[CR32] Wu J, Wang Y, Liu H, Yang H, Ma D, Li J, Li D, Lai R, Yu H (2010). Two immune-regulatory peptides with antioxidant activity from tick salivary glands. J Biol Chem.

[CR33] Wu J, Liu H, Yang H, Yu H, You D, Ma Y, Ye H, Lai R (2011). Proteomic analysis of skin defensive factors of tree frog Hyla simplex. J Proteome Res.

[CR34] Wu J, Mu L, Zhuang L, Han Y, Liu T, Li J, Yang Y, Yang H, Wei L (2015). A cecropin-like antimicrobial peptide with anti-inflammatory activity from the black fly salivary glands. Parasit Vectors.

[CR35] Xu X, Lai R (2015). The chemistry and biological activities of peptides from amphibian skin secretions. Chem Rev.

[CR36] Yang H, Wang X, Liu X, Wu J, Liu C, Gong W, Zhao Z, Hong J, Lin D, Wang Y, Lai R (2009). Antioxidant peptidomics reveals novel skin antioxidant system. Mol Cell Proteomics.

[CR37] Yu H, Cai S, Gao J, Zhang S, Lu Y, Qiao X, Yang H, Wang Y (2013). Identification and polymorphism discovery of the cathelicidins, Lf-CATHs in ranid amphibian (*Limnonectes fragilis*). FEBS J.

[CR38] Zanetti M (2005). The role of cathelicidins in the innate host defenses of mammals. Curr Issues Mol Biol.

