# Supplementary material for: The first identified cathelicidin from tree frogs possesses anti-inflammatory and partial LPS neutralization activities
Source: Amino Acids. 2017 Jun 7;49(9):1571–85. doi: 10.1007/s00726-017-2449-7 (PMC5561178; doi:10.1007/s00726-017-2449-7)
Supplement: Supplementary file 1 — Supplementary material 1 (DOC 38 kb) [file 726_2017_2449_MOESM1_ESM.doc]

**Table S1.** Primer sequences used for cloning and qPCR in this study

| **Primer** | **Sequence (5’→3’)** | **application** |
| --- | --- | --- |
| Cathelicidin-PP -R_1_ | CAIARIARRTTRCAYTTNCCRTT* | 5’ end screening |
| 5’ PCR primer | AAGCAGTGGTATCAACGCAGAGT | 5’ end screening |
| Cathelicidin-PP-F_1_ | TCTTTCTAGCGGCTCTAACATTG | 3’ end screening |
| 3’ PCR primer | CGGGGTACGATGAGACACCA | 3’ end screening |
| Cathelicidin-PP -F_2_ | TTTAATCAAGGAGACGGAGTGCC | qPCR |
| Cathelicidin-PP -R_2_ | GACTGACGCATTCCACCTCCTCT | qPCR |
| Actin-F | CCTTCTACAATGAGCTGCGTGTT | qPCR |
| Actin-R | TACGACCAGAGGCATACAGGGAC | qPCR |
| TNF-α-F | CGGTGCCTATGTCTCAGCCT | qPCR |
| TNF-α-R | GAGGGTCTGGGCCATAGAAC | qPCR |
| IL-1β-F | ATGGCAACTGTTCCTGAACTC | qPCR |
| IL-1β-R | GCCCATACTTTAGGAAGACA | qPCR |
| IL-6-F | AGTTGCCTTCTTGGGACTGA | qPCR |
| IL-6-R | TCCACGATTTCCCAGAGAAC | qPCR |
| iNOS-F | CTGCAGCACTTGGATCAGGAACCTG | qPCR |
| iNOS -R | GGAGTAGCCTGTGTGCACCTGGAA | qPCR |

*****Where R stands for A or G; Y stands for C or T; N stands for A, C, G or T; and I stands for hypoxanthine.
